# Video games as tools to achieve insight into cognitive processes

**DOI:** 10.3389/fpsyg.2015.00003

**Published:** 2015-01-21

**Authors:** Walter R. Boot

**Affiliations:** Department of Psychology, Florida State UniversityTallahassee, FL, USA

**Keywords:** video games, cognitive, entertainment, cognitive processes, human cognition

Though traditionally designed for entertainment purposes, video games are increasingly being used by psychologists to aid in our understanding of skill acquisition, cognitive capacity and plasticity, development and aging, and individual differences. Work by Green and Bavelier (2003), now published over a decade ago, generated a great deal of interest by psychologists (and the general public) and an influx of researchers into this domain. However, the tradition of using video games to understand, measure, and improve cognition goes back to at least the mid-to-late 1980s (e.g., Griffith et al., 1983; Gagnon, 1985; Metalis, 1985; Dorval and Pepin, 1986; Clark et al., 1987). The most notable, systematic attempt to use a video game to understand human cognition and performance was the Learning Strategies Program, funded by the Defense Advanced Research Projects Agency (Donchin et al., 1989). Out of this project came the Space Fortress video game, which was developed by cognitive psychologists and designed to require skills such as memory, attention, dual-tasking ability, and psychomotor control and speed. Space Fortress served as a standardized task so performance could be compared across labs (and across continents) to understand the best methods to train complex skill, the relationship between fundamental abilities (e.g., fluid intelligence) and skill development, and the degree to which training gains and task mastery transfer beyond the trained task. Space Fortress is still used across many labs today (e.g., Blumen et al., 2010; Lee et al., 2012; Scheldrup et al., 2014), and has been one of the few video games to be played while functional magnetic resonance imaging has been recorded (e.g., Voss et al., 2012).

Space Fortress has served as an invaluable research tool, and has revealed a great deal about the nature of skill acquisition and learning. However, as anyone who has played the game can attest, Space Fortress gameplay can be a frustrating and unenjoyable experience. Space Fortress was developed by psychologists, not professional game developers. Although it was similar to many arcade games at the time of its development, by today's standards Space Fortress is a relatively primitive game. It features rudimentary graphics, it lacks an engaging narrative, its level of difficulty does not adapt to the player's skill, and it is essentially the same “level” presented to the player over and over again (there are no new challenges or game elements as the player spends more time playing the game). Modern video games feature realistic graphics, compelling stories, are adaptive, have changing demands, and allow the player to approach in-game problems in many different ways. They are designed to be motivating and challenging, but not so challenging as to arouse a high level of frustration. While many of these changes make the study of modern video games more appealing and interesting, the increased complexity and diversity of these games make performance within them more difficult to understand, and this introduces challenges when using video games as tools to achieve insight into cognitive processes. This Research Topic, containing 10 articles, and featuring 45 authors, highlights the promise and challenge of using commercial and custom video games to understand cognition.

Many of the articles included in this Research Topic revolve around the theme of transfer of training from video games to other measures of perception and cognition, inspired by the now seminal work of Shawn Green, Daphne Bavelier, and others (Bavelier et al., 2012). This research suggests that action video game play is associated with superior perceptual and cognitive abilities. Cain et al. (2014) and Pohl et al. (2014) in this Research Topic present evidence in favor of cross-sectional differences between action gamers and non-gamers on measures of vision and attention. However, evidence from cross-sectional and training studies used to support action game effects has been criticized for a variety of methodological reasons (Boot et al., 2011, 2013b; Kristjánsson, 2013; Bisoglio et al., 2014; Ferguson, 2014). Importantly, Cain et al. (2014) and Pohl et al. (2014) provide a full report of their methods and the ways in which participants were recruited, following the best reporting practices outlined by critics of game effects. In their large-sampled training study, Baniqued et al. (2013) found limited transfer of training, but blunt the potential criticism of placebo effects being responsible for the transfer effects that were observed by measuring participants' expectations regarding the type of training they received (see Blacker et al., 2014; for a similar approach).

If video game interventions are determined to improve perceptual and cognitive abilities, then they must be well-designed in order to effectively and efficiently deliver training. Montani et al. (2014) present the design and validation process used to develop a game to exercise attention and executive functioning in individuals with Traumatic Brain Injury (TBI). They demonstrate that their game does tap aspects of executive control, and future intervention studies with TBI patients as participants will determine whether game improvements transfer to other measures of executive control, and more importantly, meaningful measures of everyday performance. Boot et al. (2013a) demonstrate another important issue in terms of video game intervention design. Game intervention design needs to consider the target population of the intervention and the preferences of that population, or intervention adherence will be low and the intervention will fail.

Within this Research Topic, Towne et al. (2014) and Latham et al. (2013) raise interesting and important questions regarding how to measure and classify video game expertise. Many studies use fairly simplistic, undifferentiated definitions of game experience. These definitions often don't make distinctions between very different types of game experience (lumping most fast-paced games into the category of “action game,” even though perceptual and cognitive demands may differ greatly between these games). To truly understand the potential effects of game experience on the performance of other laboratory and real-world tasks, we need to better measure how often individuals are playing video games, what they are playing, and their history of gameplay across their lifetime. Towne et al. (2014) argue that methods from the study of expertise in other domains (e.g., chess) can serve as an example.

Finally, Ventura et al. (2013) present a different example of the way video games can be used to understand cognition. Custom games can be used as a way to measure cognitive abilities, much in the same way the Space Fortress game could be seen as a measure of fluid intelligence (Rabbitt et al., 1989). This “stealth assessment” has several advantages, including the reduction of test anxiety. This extremely promising approach might be ported to the laboratory to get better ability measures compared to our typically dull battery of intimidating neuropsychological tests.

This is truly an exciting and fast-moving area of research with many challenges, but also potentially many rewards. Fortunately, we are seeing more and more studies taking steps to overcome these challenges, and more discussion of best practices with respect to using games to gain insight into cognitive processes (including studies and discussion presented in this Research Topic).

## Conflict of interest statement

The author declares that the research was conducted in the absence of any commercial or financial relationships that could be construed as a potential conflict of interest.

## References

[B1] Baniqued P. L. Kranz M. B. Voss M. W. Lee H. Cosman J. D. Severson J.. (2013). Cognitive training with casual video games: points to consider. Front. Psychol. 4:1010. 10.3389/fpsyg.2013.01010 24432009 PMC3882717

[B2] Bavelier D. Green C. S. Schrater P. Pouget A. (2012). Brain plasticity through the life span: learning to learn and action video games. Annu. Rev. Neurosci. 35, 391–416. 10.1146/annurev-neuro-060909-152832 22715883

[B3] Bisoglio J. Michaels T. I. Mervis J. E. Ashinoff B. K. (2014). Cognitive enhancement through action video game training: great expectations require greater evidence. Front. Psychol. 5:136. 10.3389/fpsyg.2014.00136 24600427 PMC3928536

[B4] Blacker K. J. Curby K. M. Klobusicky E. Chein J. M. (2014). Effects of action video game training on visual working memory. J. Exp. Psychol. Hum. Percept. Perform. 40, 1992–2004. 10.1037/a0037556 25068696

[B5] Blumen H. M. Gopher D. Steinerman J. R. Stern Y. (2010). Training cognitive control in older adults with the space fortress game: the role of training instructions and basic motor ability. Front. Aging Neurosci. 2:145. 10.3389/fnagi.2010.00145 21120135 PMC2991174

[B6] Boot W. R. Blakely D. P. Simons D. J. (2011). Do action video games improve perception and cognition? Front. Psychol. 2:226. 10.3389/fpsyg.2011.00226 21949513 PMC3171788

[B7] Boot W. R. Champion M. Blakely D. P. Wright T. Souders D. J. Charness N. (2013a). Video games as a means to reduce age-related cognitive decline: attitudes, compliance, and effectiveness. Front. Psychol. 4:31. 10.3389/fpsyg.2013.00031 23378841 PMC3561600

[B8] Boot W. R. Simons D. J. Stothart C. Stutts C. (2013b). The pervasive problem with placebos in psychology why active control groups are not sufficient to rule out placebo effects. Perspect. Psychol. Sci. 8, 445–454 10.1177/1745691613491271 26173122

[B9] Cain M. S. Prinzmetal W. Shimamura A. P. Landau A. N. (2014). Improved control of exogenous attention in action video game players. Front. Psychol. 5:69. 10.3389/fpsyg.2014.00069 24575061 PMC3918660

[B10] Clark J. E. Lanphear A. K. Riddick C. C. (1987). The effects of videogame playing on the response selection processing of elderly adults. J. Gerontol. 42, 82–85. 10.1093/geronj/42.1.82 3794204

[B11] Donchin E. Fabiani M. Sanders A. (eds.). (1989). The learning strategies program: an examination of the strategies in skill acquisition [Special issue]. Acta Psychol. 71, 1–309. 10.1016/0001-6918(89)90002-4 2816473

[B12] Dorval M. Pepin M. (1986). Effect of playing a video game on a measure of spatial visualization. Percept. Mot. Skills 62, 159–162. 10.2466/pms.1986.62.1.159 3960656

[B13] Ferguson C. J. (2014). Action game experimental evidence for effects on aggression and visuospatial cognition: similarities, differences, and one rather foolish question. Front. Psychol. 5:88. 10.3389/fpsyg.2014.00088 24570673 PMC3916832

[B14] Gagnon D. (1985). Videogames and spatial skills: an exploratory study. Educ. Commun. Technol. J. 33, 263–275.

[B15] Green C. S. Bavelier D. (2003). Action video game modifies visual selective attention. Nature 423, 534–537. 10.1038/nature01647 12774121

[B16] Griffith J. L. Voloschin P. Gibb G. D. Bailey J. R. (1983). Differences in eye-hand motor coordination of video-game users and non-users. Percept. Mot. Skills 57, 155–158. 10.2466/pms.1983.57.1.155 6622153

[B17] Kristjánsson Á. (2013). The case for causal influences of action videogame play upon vision and attention. Attent. Percept. Psychophys. 75, 667–672. 10.3758/s13414-013-0427-z 23386038

[B18] Latham A. J. Patston L. L. M. Tippett L. J. (2013). Just how expert are “expert” video-game players? Assessing the experience and expertise of video-game players across “action” video-game genres. Front. Psychol. 4:941. 10.3389/fpsyg.2013.00941 24379796 PMC3863720

[B19] Lee H. Boot W. R. Basak C. Voss M. W. Prakash R. S. Neider M.. (2012). Performance gains from directed training do not transfer to untrained tasks. Acta Psychol. 139, 146–158. 10.1016/j.actpsy.2011.11.003 22133724

[B20] Metalis S. A. (1985). Effects of massed versus distributed practice on acquisition of video game skill. Percept. Mot. Skills 61, 457–458 10.2466/pms.1985.61.2.457

[B21] Montani V. De Grazia M. D. F. Zorzi M. (2014). A new adaptive videogame for training attention and executive functions: design principles and initial validation. Front. Psychol. 5:409. 10.3389/fpsyg.2014.00409 24860529 PMC4026745

[B22] Pohl C. Kunde W. Ganz T. Conzelmann A. Pauli P. Kiesel A. (2014). Gaming to see: action video gaming is associated with enhanced processing of masked stimuli. Front. Psychol. 5:70. 10.3389/fpsyg.2014.00070 24550879 PMC3913992

[B23] Rabbitt P. Banerji N. Szymanski A. (1989). Space Fortress as an IQ test? Predictions of learning and of practised performance in a complex interactive video-game. Acta Psychol. 71, 243–257 10.1016/0001-6918(89)90011-5

[B24] Scheldrup M. Greenwood P. M. McKendrick R. Strohl J. Bikson M. Alam M.. (2014). Transcranial direct current stimulation facilitates cognitive multi-task performance differentially depending on anode location and subtask. Front. Hum. Neurosci. 8:665. 10.3389/fnhum.2014.00665 25249958 PMC4157612

[B25] Towne T. J. Ericsson K. A. Sumner A. M. (2014). Uncovering mechanisms in video game research: suggestions from the expert-performance approach. Front. Psychol. 5:161. 10.3389/fpsyg.2014.00161 24624103 PMC3939770

[B26] Ventura M. Shute V. Wright T. Zhao W. (2013). An investigation of the validity of the virtual spatial navigation assessment. Front. Psychol. 4:852. 10.3389/fpsyg.2013.00852 24379790 PMC3861692

[B27] Voss M. W. Prakash R. S. Erickson K. I. Boot W. R. Basak C. Neider M. B.. (2012). Effects of training strategies implemented in a complex videogame on functional connectivity of attentional networks. Neuroimage 59, 138–148. 10.1016/j.neuroimage.2011.03.052 21440644

